# Shorter sleep duration is associated with potential risks for overwork-related death among Japanese truck drivers: use of the Karoshi prodromes from worker’s compensation cases

**DOI:** 10.1007/s00420-021-01655-5

**Published:** 2021-02-01

**Authors:** Tomohide Kubo, Shun Matsumoto, Takeshi Sasaki, Hiroki Ikeda, Shuhei Izawa, Masaya Takahashi, Shigeki Koda, Tsukasa Sasaki, Kazuhiro Sakai

**Affiliations:** 1 National Institute of Occupational Safety and Health, 6-21-1, Nagao, Tama-ku, Kawasaki, 214-8585 Japan; 2 Ohara Memorial Institute for Science of Labour, Tokyo, Japan

**Keywords:** Excessive fatigue, Karoshi, Overtime, Overwork-related cerebrovascular and cardiovascular diseases, Sleep, Recovery from fatigue, Working hours

## Abstract

**Purpose:**

We aimed to cross-sectionally investigate how work and sleep conditions could be associated with excessive fatigue symptoms as an early sign of Karoshi (overwork-related cerebrovascular and cardiovascular diseases; CCVDs).

**Methods:**

We distributed a questionnaire regarding work, sleep, and excessive fatigue symptoms to 5410 truck drivers, as the riskiest occupation for overwork-related CCVDs, and collected 1992 total samples (response rate: 36.8%). The research team collected 1564 investigation reports required for compensation for Karoshi. Of them, 190 reports listed the prodromes of Karoshi, which were used to develop the new excessive fatigue symptoms inventory.

**Results:**

One-way analyses of variance showed that the excessive fatigue symptoms differed significantly by monthly overtime hours (*p* < 0.001), daily working time (*p* < 0.001), work schedule (*p* = 0.025), waiting time on-site (*p* = 0.049), number of night shifts (*p* = 0.011), and sleep duration on workdays (*p* < 0.001). Multivariate mixed-model regression analyses revealed shorter sleep duration as the most effective parameter for predicting excessive fatigue symptoms. Multiple logistic regression analysis confirmed that the occurrences of CCVDs were significantly higher in the middle [adjusted ORs = 3.56 (1.28–9.94)] and high-score groups [3.55 (1.24–10.21)] than in the low-score group.

**Conclusion:**

The findings suggested that shorter sleep duration was associated more closely with a marked increase in fatigue, as compared with the other work and sleep factors. Hence, ensuring sleep opportunities could be targeted for reducing the potential risks of Karoshi among truck drivers.

**Supplementary Information:**

The online version contains supplementary material available at 10.1007/s00420-021-01655-5.

## Introduction

In the short run, fatigue at work could lead to occupational injuries when workers could not sufficiently recover from fatigue (Swaen et al. 2003). In the long run, incomplete recovery from fatigue could be a potential risk for diseases (Van Amelsvoort et al. 2003). According to the effort-recovery model (Meijman and Mulder 1998), recovery occurs when work demands no longer strain an individual’s resources. Meanwhile, fatigue could progress to excessive fatigue linked to disease when one’s recovery periods are constantly deprived by overwork. However, empirical evidence is still lacking to understand whether excessive fatigue could cause death or disorders such as Karoshi [i.e., death or disorder by overwork-related cerebrovascular/cardiovascular diseases (CCVDs)] (The Government of Japan 2014).

The Japanese word “Karoshi” has been internationally recognized since it was listed in the Oxford English Dictionary in 2002 (North and Morioka 2016). Approximately 250 Karoshi cases are compensated annually (Japan Ministry of Health, Labour, and Welfare 2020). This problem has spread throughout other countries, especially in Asian countries, during the last decade (Cheng et al. 2012; Lin et al. 2019; Yang et al. 2019). In addition, we expect that essential workers, such as health care workers, could be suffering from overwork due to COVID-19 (Chen et al. 2020; He et al. 2020). In 2014, the Japanese government established a new law to take preventive measures against the Karoshi problems (Japan Ministry of Health, Labour, and Welfare 2016). Moreover, the law stipulates that the Japanese national institute conducts research to analyze the mechanisms of Karoshi by collecting worker’s compensation claims for overwork from all areas of Japan.

According to an earlier study, about two-thirds of the victims in a sample of 203 cases showed long working hours (more than 60 h per week) and excessive overtime (more than 50 h per month), and half of them worked on their day off (Uehata 1991). Therefore, long working hours is one of the major factors of Karoshi. A previous systematic review also showed that employees who work long hours have a higher risk of stroke than those who work standard hours, while the association is weaker for coronary heart disease (Kivimäki et al. 2015). On the other hand, the definition of long working hours ranged between 45 and 60 h per week in the review. Given that long working hours in relation to Karoshi is defined as more than 80 h per week in Japan (Japan Ministry of Health, Labour, and Welfare 2016), more empirical data are still needed to understand the pathway between excessive long working hours (i.e., more than 80 working hours) and Karoshi.

Furthermore, a previous study suggested a U-shaped association between working hours and the risk of acute myocardial infarction in their case–control study (Sokejima and Kagamimori 1998). Contrary to the expectations, the findings suggested that employees who did not work long hours could have higher risks of Karoshi. In other words, potential factors, other than long working hours, likely could be linked to the occurrence of Karoshi. Indeed, several cases without overtime were compensated as Karoshi, according to a governmental report (Japan Ministry of Health, Labour, and Welfare 2020).

Earlier studies have been relatively focused on stressors (i.e., external factors) such as working hours. However, an earlier study reported that employees with high job strain and effort–reward imbalance had higher risks of cardiovascular mortality than employees without them did (Kivimäki et al. 2002). Namely, available data regarding strains (i.e., internal factors) are limited. Hence, it is informative to understand how employees feel exhausted until the onset of Karoshi. Detecting specific symptoms in response to overwork is vital to prevent the problems. For instance, vital exhaustion—a state defined as unusual fatigue, loss of energy, increased irritability, and feelings of demoralisation—may be one of the candidate indicators to predict the risk of Karoshi (Meesters and Appels 1996). However, the finding of vital exhaustion has often targeted patients, not employees. In addition, vital exhaustion is considered as an earlier sign of cardiovascular disease, not including cerebrovascular diseases. As far as we know, no appropriate tool exists with which to evaluate the risks of Karoshi in relation to work-related factors.

To fill the gap, we used data on Karoshi prodromes listed in investigation reports for worker’s compensation claims to develop a new questionnaire for evaluating symptoms of excessive fatigue. Then we focused on truck drivers because they are the most numerous occupation among Karoshi cases, according to a governmental report (Japan Ministry of Health, Labour, and Welfare 2020). This study is aimed at investigating how work-related factors could be associated with excessive fatigue symptoms as an earlier sign of Karoshi. Based on the findings, we explored potential preventive measures against Karoshi. Moreover, we examined the association between excessive fatigue symptoms, as measured by the newly developed questionnaire, and medical history of CCVDs to test the predictability of Karoshi risks.

## Methods

### Study sample

We obtained a list of truck driver companies from the Japan Trucking Association (JTA), which is the biggest professional drivers’ organization in Japan (Affiliated; 62,905 companies). The list describes information regarding the affiliated company’s size and working style. To avoid selection bias, we requested the branch chiefs, who affiliate JTA in each area, to select the possible respondents while considering the same proportions based on the company’s size (≥ 50 or < 50 employees) and the working style (long or short haul). Then we distributed a questionnaire regarding work, sleep, and excessive fatigue symptoms to 5410 truck drivers from 1082 companies in all 47 prefectures of Japan. Consequently, 1992 drivers answered the questionnaire (response rate: 36.8%). The Institutional Review Board of the National Institute of Occupational Safety and Health reviewed and approved the study protocol (H2824).

### Excessive fatigue symptom inventory

Our research team collected 1564 documents from all areas of Japan, which were accepted to compensate Karoshi cases from January 2010 through March 2015. Of them, 190 documents listed the prodromes of Karoshi, which were used to develop the new Excessive Fatigue Symptom Inventory (EFSI). A labor standards inspector recorded those prodromes based on a medical certificate or an interview from the victim’s family. First, we condensed a sentence into a short word regarding excessive fatigue symptoms, and thereby 162 words were derived from 190 documents. After that, we categorized the prodromes by similar symptoms with the K–J method (Scupin 1997). Then one of the co-authors (SK), who is a medical doctor, provided medical advice on whether the prodromes could be related to excessive fatigue symptoms. In addition, previous research conducted interviews with the family of Karoshi victims and reported some excessive fatigue symptoms before the onset of Karoshi (Saito 1993). Given the medical advice and the previous finding, those prodromes were classified into 26 excessive fatigue symptoms: (1) abnormal sweat, (2) severe back and shoulder pain, (3) face flushing, (4) chest pain and oppressive feeling, (5) breathing difficulties, (6) repeated vomiting, (7) heart palpitation, (8) arm and foot numbness, (9) sudden blindness, (10) heavy headache and dizziness, (11) slurring words, (12) heavy toothache, (13) emotionally arguing with someone, (14) sudden unconsciousness, (15) unstoppable nosebleed, (16) difficulty falling asleep at night, (17) significant weight loss, (18) unrecoverable abnormal fatigue regardless of sleeping or resting, (19) abnormal sleepiness, (20) having a short fuse, (21) losing one’s appetite, (22) frequently thinking about quitting one’s job, (23) spending one’s days off sleeping, (24) going to bed immediately after work due to exhaustion, (25) difficulty awakening from sleep, and (26) becoming unable to perform daily activities. Of these 26 symptoms, 16 items were physical symptoms (i.e., the first 16 symptoms), while 10 items were behavioral symptoms (i.e., the last 10 symptoms). The respondents rated how much they experienced each symptom in the past 6 months on a 4-point scale (1 = never, 4 = always). The total score was analyzed (Cronbach’s alpha = 0.87). EFSI is available in the electronic supplementary material for this article.

### Work-related factors

We distributed a self-reported questionnaire—the EFSI—asking about the employees’ work-related factors with Karsohi risks. The questionnaire included monthly overtime, daily working hours, work schedules, waiting time on-site, number of night shifts, sleep duration, and respondent background information. Furthermore, we asked the respondents to select their monthly overtime hours in the last 3 months among the following six groups: (1) 0–20 h, (2) 21–40 h, (3) 41–60 h, (4) 61–80 h, (5) 81–100 h, or (6) > 100 h. Regarding daily working hours, we asked the respondents, “How many hours did you work in the last month?” Afterward, we categorized the respondents into the following fifth groups: (1) ≤ 8 h, (2) 9–10 h, (3) 11–12 h, (4) ≥ 13 h. Regarding work schedules, we asked the respondents to select from the following five schedules: (1) day trips, (2) day trips starting [or ending] between 22:00 and 5:00, (3) two-day trips, (4) three or four-day trips, or (5) ≥ five-day trips. Because Japanese truck drivers often have to wait to deliver their cargo to the destinations, we asked about the usual waiting time with a single question: “On average, how many hours do you wait until delivering your luggage in the destination?” We categorized the respondents into five groups: (1) never, (2) 1–2 h, (3) 3–4 h, (4) 5–6 h, or (5) ≥ 7 h. We assessed the number of night shifts in the last month with one question: “How many night shifts did you work in the last month?” In addition, we categorized the respondents into the following three groups: (1) never, (2) < 15 days, and (3) ≥ 15 days. We measured sleep duration with a single question: “How many hours did you sleep in the last month?” Consequently, we categorized the respondents into the following five groups: (1) ≥ 8 h, (2) 7 h, (3) 6 h, (4) 5 h, or (5) ≤ 4 h. Regarding the respondents’ backgrounds, we asked about their ages, genders, weight, height, habits (cigarettes, alcohol, and exercise), and job tenure. Then, we asked the respondents about their previous medical histories, including cerebrovascular disease, cardiovascular disease, hypertension, hyperlipidemia, diabetes mellitus, and obesity.

### Data analysis

Because the EFSI is a primary outcome of this study, we excluded from the data set in each statistical analysis if more than one item was missing in the EFSI. We conducted an analysis of variance (ANOVA) with Bonferroni post hoc test to examine how work-related factors could be associated with the EFSI. Afterward, we conducted a multivariate mixed-model regression to reveal which work-related factors could have stronger effects on the total EFSI score among the six work-related factors. The covariates included age, gender, BMI, alcohol, smoking, exercise, and job tenure. Finally, we divided the EFSI scores into three groups [i.e., low (reference), middle, and high] according to the tertile. We calculated crude odds ratios (ORs) to examine the association between the excessive fatigue score and medical history of CCVDs through a multiple logistic regression analysis. In addition, we tested three models to determine how the association could be influenced by the respondents’ background (model 2: age, gender, alcohol, smoking, and exercise habits) or work-related factors (model 3: overtime, night shift, working hours, sleep duration, wait times on-site, work schedule, and job tenure) or Karoshi-related diseases (model 4: hypertension, hyperlipidemia, and diabetes mellitus). We conducted the same analyses in the medical history of hypertension, hyperlipidemia, and diabetes mellitus because those diseases are regarded as a potential trigger for Karoshi. We performed all of the statistical analyses using IBM SPSS Statistics version 26 (IBM Corp., Armonk, NY, USA).

## Results

### Respondent characteristics

As shown in Table 1, the respondents were 97.7% male, and their mean age, body mass index, and job tenure years were 46.4 years [standard deviation (SD): 9.1], 24.3 (SD: 3.8), and 18.8 (SD: 10.4), respectively. Regarding habits, a majority of the respondents smoked cigarettes (53.3%), drank alcohol (67.1%), and did not exercise (80.6%). The top three diseases in medical histories were obesity (21.9%), hypertension (19.1%), and hyperlipidemia (8.3%). The mean and SD of work-related factors were as follows: daily working hours—14.4 h (SD: 13.3 h), waiting time on-site—1.6 h (SD: 3.4), and number of night shifts per month—5.5 h (SD: 7.5). In addition, the highest proportions of respondents had 0–20 h of monthly overtime (34.3%) and a day-trip schedule (59.4%).

**Table 1:** Characteristics of the respondents

*N* = 1992	No. of respondents	%	Mean	SD
Gender				
Male	1947	97.7		
Female	41	2.1		
Missing	4	0.2		
Age (years)			46.4	9.1
BMI			24.3	3.8
Job tenure (years)			18.8	10.4
Cigarette				
No	357	17.9		
Yes (in the past)	563	28.3		
Yes	1062	53.3		
Missing	10	0.5		
Alcohol				
Yes	1336	67.1		
No	648	32.5		
Missing	8	0.4		
Exercise				
Yes	382	19.2		
No	1605	80.6		
Missing	5	0.3		
Medical history				
Cerebrovascular disease	14	0.7		
Cardiovascular disease	48	2.4		
Hypertension	381	19.1		
Hyperlipidemia	166	8.3		
Diabetes	110	5.5		
Obesity	437	21.9		
Daily working times (hours)			14.4	13.3
Waiting time on-site (hours)			1.6	3.4
No. of night shifts (/month)			5.5	7.5
Monthly overwork hours				
0–20 h	684	34.3		
21–40 h	410	20.6		
41–60 h	350	17.6		
61–80 h	224	11.2		
81–100 h	98	4.9		
> 100 h	76	3.8		
Missing	150	7.5		
Work schedule				
Day trip	1183	59.4		
Day trip (22 pm–5 am)	269	13.5		
2-day trips	278	14.0		
3- or 4-days trip	176	8.8		
≥ 5-day trips	47	2.4		
Missing	39	2.0		

### Work-related factors and excessive fatigue score

**Fig. 1:**
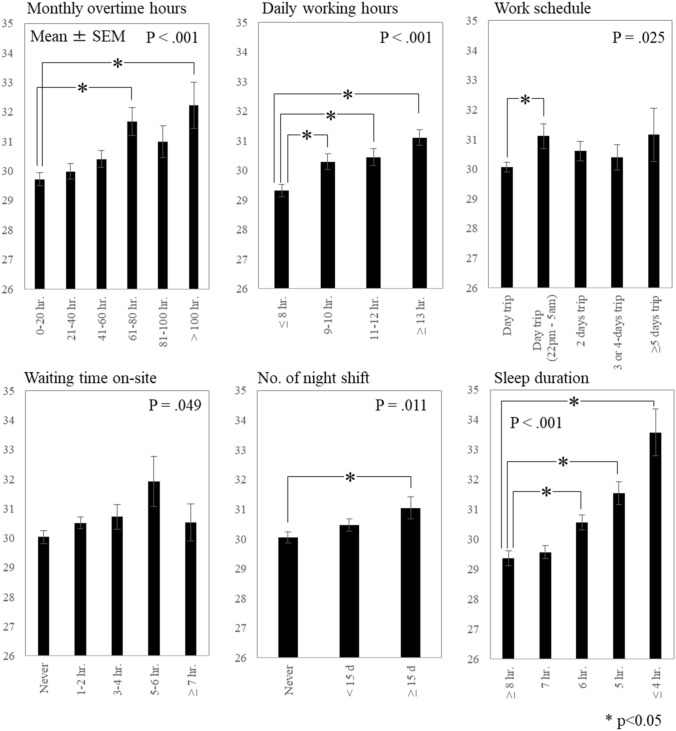
Association between work-related factors and Excessive Fatigue Symptom Inventory score

Figure 1 indicates the total EFSI scores according to work-related factors. We found significant differences in the factors of monthly overtime hours (*p* < 0.001), daily working hours (*p* < 0.001), work schedule (*p* = 0.025), waiting time on-site (*p* = 0.049), number of night shifts (*p* = 0.011), and sleep duration (*p* < 0.001). In particular, significantly higher EFSI scores were observed for those with longer hours of overtime and daily working times, greater number of night shifts, and shorter sleep durations. Regarding work schedule, a post hoc test showed that the EFSI score was significantly higher among the day trip [starting (or ending) between 22:00 and 5:00] group than in the day trip group (*p* < 0.05).

### Predicting excessive fatigue score from work-related factors

Table 2 shows the results from the multivariate mixed-model regression for predicting an excessive fatigue score. We entered and thereby adjusted all of the work-related factors at the same time. The excessive fatigue score was significantly related to monthly overtime hours, work schedule, waiting time on-site, and sleep duration. In particular, excessive fatigue was strongly correlated with sleep duration, increasing by 3.8 units (beta coefficient) for the ≤ 4-h group, 2.5 units for the 5-h group, and 1.5 units for the 6-h group, compared with the ≥ 8-h (reference) group. It was also related to monthly overtime hours, increasing 1.3 units for those with 61–80 h of overtime, compared with those working 8 or more hours of overtime. Excessive fatigue increased by 1.1 units for the group taking trips lasting 5 or more days, compared with those having day trip work schedules, and it increased by 2.5 units for the group with 5–6 h of waiting time on-site, compared with the reference group.

**Table 2:** Coefficients of linear regression for the Excessive Fatigue Symptom Inventory

Parameters	*N*	*Β*	SE	DF	*t*	*p*	95% CI
Intercept		**24.877**	**1.964**	**1487**	**12.7**	** < 0.001**	**21.0/28.7**
Monthly overtime hours							
> 100 h	76	1.568	0.806	1487	1.9	0.052	0.0/3.1
81–100 h	98	0.368	0.676	1487	0.5	0.586	− 1/1.7
61–80 h	224	**1.299**	**0.487**	**1487**	**2.7**	**0.008**	**0.3/2.3**
41–60 h	350	0.239	0.411	1487	0.6	0.561	− 6/1.0
21–40 h	410	− 0.039	0.384	1487	− 0.1	0.918	− 0.8/0.7
0–20 h	684	Ref	−	−	−	−	−
Daily working hours							
≥ 13 h	605	− 0.165	0.512	1487	− 0.3	0.747	− 1.2/0.8
11–12 h	444	0.583	0.479	1487	1.2	0.224	− 0.4/1.5
9–10 h	508	0.142	0.451	1487	0.3	0.753	− 0.7/1.0
≤ 8 h	435	Ref	−	−	−	−	−
Work schedule							
≥ 5-day trip	47	**2.807**	**1.081**	**1487**	**2.6**	**0.009**	**0.7/4.9**
3- or 4-day trip	176	− 0.149	0.648	1487	− 0.2	0.819	− 1.4/1.1
2-Day trip	278	0.464	0.532	1487	0.9	0.384	− 0.6/1.5
Day trip (22 pm–5 am)	269	0.667	0.495	1487	1.3	0.178	− 0.3/1.6
Day trip	1183	Ref	–	–	–	–	–
Waiting time on–site							
≥ 7 h	67	0.134	0.824	1487	0.2	0.871	− 1.5/1.7
5–6 h	60	**2.452**	**0.855**	**1487**	**2.9**	**0.004**	**0.8/4.1**
3–4 h	185	0.098	0.546	1487	0.2	0.857	− 1.0/1.2
1–2 h	889	0.291	0.318	1487	0.9	0.360	− 0.3/0.9
Never	653	Ref	–	–	–	–	–
No. of night shifts							
≥ 15 days	361	− 0.166	0.500	1487	− 0.3	0.740	− 1.1/0.8
< 15 days	689	0.114	0.379	1487	0.3	0.763	− 0.6/0.9
Never	942	Ref	–	–	–	–	–
Sleep duration							
≤ 4 h	106	**3.841**	**0.682**	**1487**	**5.6**	** < 0.001**	**2.5/5.2**
5 h	291	**2.502**	**0.483**	**1487**	**5.2**	** < 0.001**	**1.6/3.4**
6 h	476	**1.451**	**0.416**	**1487**	**3.5**	** < 0.001**	**0.6/2.3**
7 h	537	0.492	0.402	1487	1.2	0.222	− 0.3/1.3
≥ 8 h	394	Ref	–	–	–	–	–

Covariates: age, gender, BMI, alcohol, smoking, exercise, and job tenure. Values in bold indicate significant differences

### Excessive fatigue symptoms and Karoshi-related diseases

As shown in Table 3, the occurrences of CCVDs were significantly higher in the middle [Crude ORs = 2.93 (95% CI; 1.31–6.51)] and high groups [Crude OR = 2.87 (1.28–6.43)] than in the low group (reference). After adjusting for life habit variables, the association between excessive fatigue and CCVDs was stronger than in the bivariate model [middle group = 3.47 (1.31–6.51); high group = 3.85 (1.28–6.43)]. Moreover, the association was much stronger than adjustment model 2, after controlling for the work-related variables [middle group = 3.85 (1.40–10.58); high group = 4.45 (1.58–12.54)]. However, after adjusting for Karoshi-related diseases (i.e., hypertension, hyperlipidemia and diabetes mellitus), the association was somewhat weaker [middle group = 3.56 (1.28–9.94); high group = 3.55 (1.24–10.21)] than adjustment model 3. Regarding hypertension, we observed no significant associations in models 1 and 2. However, the occurrences of hypertension were significantly higher in the middle [1.49 (1.04–2.12)] and high groups [1.60 (1.11–2.32)] than in the low group in model 3. On the other hand, the associations between excessive fatigue of hyperlipidemia and diabetes mellitus were significant in model 2, while model 1 did not show a significant difference. Namely, the occurrences of hyperlipidemia and diabetes mellitus were significantly higher in the high group than in the low group [1.59 (1.04–2.42) and 1.93 (1.11–3.33), respectively]. Moreover, we observed significantly stronger associations in the high group after adjusting for all variables on model 3 [1.80 (1.10–2.93) and 2.40 (1.28–4.49), respectively].

**Table 3:** Association between Excessive Fatigue Symptom Inventory and Karoshi-related diseases

Excessive Fatigue Symptom Inventory score (26–104)	Diseases	No diseases	Model 1^a^		Model 2^b^		Model3^c^		Model 4^d^	
			Crude OR	95% CI	Adjusted OR	95% CI	Adjusted OR	95% CI	Adjusted OR	95% CI
CCVDs										
Low (less than 27)	8	597	Ref		Ref		Ref		Ref	
Middle (27–30)	26	663	**2.93**	**1.31, 6.51**	**3.47**	**1.31, 6.51**	**3.85**	**1.40, 10.58**	**3.56**	**1.28, 9.94**
High (greater than 30)	24	625	**2.87**	**1.28, 6.43**	**3.85**	**1.28, 6.43**	**4.45**	**1.58, 12.54**	**3.55**	**1.24, 10.21**
Hypertension										
Low (less than 27)	102	503	Ref		Ref		Ref			
Middle (27–30)	142	547	1.28	0.97, 1.70	1.30	0.96, 1.76	**1.49**	**1.04, 2.12**		
High (greater than 30)	124	525	1.16	0.87, 1.56	1.26	0.91, 1.72	**1.60**	**1.11, 2.32**		
Hyperlipidemia										
Low (less than 27)	44	561	Ref		Ref		Ref			
Middle (27–30)	54	635	1.08	0.72, 1.64	1.14	0.75, 1.75	1.18	0.72, 1.92		
High (greater than 30)	64	585	1.39	0.93, 2.08	**1.59**	**1.04, 2.42**	**1.80**	**1.10, 2.93**		
Diabetes mellitus										
Low (less than 27)	26	579	Ref		Ref		Ref			
Middle (27–30)	38	651	1.30	0.78, 2.17	1.41	0.82, 2.42	1.62	0.87, 3.00		
High (greater than 30)	41	608	1.50	0.90, 2.49	**1.93**	**1.11, 3.33**	**2.40**	**1.28, 4.49**		

Values in bold indicate significant differences

*CCVDs* cerebrovascular and cardiovascular diseases, *OR* odds ratio, *CI* confidential interval

^a^Adjustment: Model 1—bivariate model without any covariates

^b^Adjustment: Model 2—model 1 + age, gender, alcohol, smoking, and exercise

^c^Adjustment: Model 3—model 2 + overtime, night shift, working hours, sleep duration, waiting time on-site, work schedule, and job tenure

^d^Adjustment: Model 4—model 3 + hypertension, hyperlipidemia, and diabetes mellitus

## Discussion

In this study, we examined the cross-sectional association between work- and sleep-related factors and excessive fatigue symptoms as an early sign of Karoshi in 1992 truck drivers by using the EFSI. At the same time, we tested whether the EFSI could be linked to the occurrence of CCVDs. Consequently, all work-related factors showed significant associations with excessive fatigue in the univariate relations (Fig. 1). However, given that we entered and adjusted all of the work-related factors, shorter sleep duration could be strongly associated with increased excessive fatigue, compared with the other work-related factors (Table 2). In addition, a higher excess fatigue score was linked to the occurrence of CCVDs, even when adjusting some covariates (Table 3). We observed the same significant associations in hypertension, hyperlipidemia, and diabetes mellitus.

As mentioned above, the law strictly limits the national institute’s use of worker’s compensation data (The Government of Japan 2014). Hence, this is the first study to examine systematically the association between work-related factors and the actual symptoms linked to Karoshi cases. On the other hand, earlier research suggests that vital exhaustion measured by the 21-item Maastricht Questionnaire could predict the occurrence of heart-related diseases (Williams et al. 2010). Because the EFSI is aimed at predicting the potential risks of Karoshi cases, the questionnaire on vital exhaustion is thought to have a similar concept to that of the EFSI. However, the item content does not overlap between the Vital Exhaustion Questionnaire and EFSI. Namely, while vital exhaustion is somewhat linked to mental state (e.g., depression), many symptoms of the EFSI are derived from physical states linked to disease. Of the EFSI items, chest pain, vomiting, sweating, neck or back pain, and shortness of breath are recognized as symptoms of a heart attack, while severe headache, dizziness, sudden vision loss, and loss of consciousness are recognized as symptoms of a stroke (The British National Health Service 2020). Furthermore, the EFSI items include some critical symptoms related to sleep loss, stress, and depression. Meanwhile, vital exhaustion could be associated with cardiovascular diseases, rather than with cerebrovascular diseases. Therefore, the EFSI may be an effective tool to assess broadly the potential risks of Karoshi cases, including cardiovascular/cerebrovascular diseases and mental disorders.

Notably, the EFSI was more strongly associated with sleep duration than with other relevant factors. Specifically, the EFSI score significantly increased by about four units (beta = 3.8) with 4 h or less of sleep duration, compared with having 8 h or more of sleep (see Table 2). Meanwhile, our initial hypothesis was that the impacts of night shifts, overtime, and daily working hours on excessive fatigue would be higher than shown here. However, these findings are in line with a finding from the meta-analysis, showing that the effect size for occupational fatigue was larger for sleep deprivation than for long working hours and overtime (Techera et al. 2016). Moreover, because short sleepers (less than 5 h per night) were reported to have a 12% higher risk of mortality than those sleeping 7–8 h per night in a systematic review (Cappuccio et al. 2010), it is reasonable that sleep plays critical roles in recovery from fatigue and stress (Härmä 2006). In addition, earlier research with 260 long-haul truck drivers suggested that good sleep quality could reduce increasing cardiovascular disease risk (Hege et al. 2018). Based on those findings, scheduling working hours coupled with sufficient sleep opportunities could be a primary measure against Karoshi cases among truck drivers. For instance, ensuring intervals between shifts (Ikeda et al. 2017; Kubo et al. 2018; Tsuchiya et al. 2017) or implementing a fatigue risk-management system (Honn et al. 2019) would be major options.

Moreover, work schedules with 5-day trips or more (beta = 2.8) and on-site waiting times of 5–6 h (beta = 2.5) were more closely related to the EFSI score in addition to sleep duration (Table 2). With regard to long-haul driving, the available data have accumulated to suggest the risks of unperceived sleepiness (Sallinen et al. 2020; Crizzle et al. 2020) and cardiovascular diseases (Lemke et al. 2017) among long-haul drivers. On the other hand, little is known about the negative influence of on-site waiting times on health outcomes among truck drivers. However, the Japan Trucking Association strongly recommends reducing the length of on-site waiting times because waiting on-site could trigger overtime or long working hours (Japan Trucking Association 2018). The reason we could not find the relevant findings may be that truck drivers waiting on-site could be a unique work issue in Japan. Namely, because Japan lacks the space to build spacious facilities that can accommodate many trucks at once (especially in urban areas), truck drivers are often required to wait on-site to deliver cargo. Thus, our findings scientifically support the above-mentioned recommendation and could be generalized to countries with similar issues.

Next, our primary question here is to answer whether the EFSI could predict the occurrence of Karoshi cases. As shown in Table 3, we found a significant association between the EFSI score and a medical history of CCVDs, which remained even after adjusting all of the covariates. Notably, the linkage was stronger in the adjusted models than in the crude model as well. Furthermore, we found the same significant associations in hypertension, hyperlipidemia, and diabetes mellitus, which have been regarded as triggers for Karoshi. These findings would partially support the predictability of Karoshi cases being evaluated by the EFSI. However, the CCVDs were retrospectively assessed by self-reported outcomes. In other words, the respondents who answered as having a medical history of CCVDs are thought to be recovered from those diseases at the time. Nonetheless, our findings show a significant linkage between current EFSI and a history of CCVDs. Then there are at least two possibilities with which to explain the data. One possibility is that previous patients with CCVDs had symptoms regarding Karoshi prodromes for a long period even after recovering from the disease. The other is that they could have higher recurrence risks of CCVDs when exposed to overwork-related factors. If the latter explanation was valid, then truck drivers who have suffered from CCVDs would be vulnerable to overwork, especially from sleep loss. Hence, organizational care should be taken to avoid overwork by employers.

Furthermore, it remains unclear whether overwork caused the medical histories of CCVDs measured by the questionnaire. Because Karoshi is defined as overwork-related death or disorders due to cerebrovascular/cardiovascular diseases (Japan Ministry of Health, Labour, and Welfare 2016), our goal is to evaluate the future risks of death or disorders due to overwork. However, a higher EFSI score was significantly related to longer overtime and working hours in univariate analyses (Fig. 1). Hence, the EFSI is likely beneficial for evaluating the risk of overwork-related death or disorders, although more prospective studies with objective data are necessary.

### Strengths and limitations

So far, scientific knowledge has been lacking with which to better understand how employees show symptoms related to excessive fatigue until the onset of Karoshi. One of the reasons is that the data from worker’s compensation cases were closed to research use. The strength of this study was that we first used the data of Karoshi prodromes listed in the investigation reports, thereby showing the association between work-related factors and Karoshi prodromes. Furthermore, given the significant associations between higher excess fatigue scores and the occurrence of Karoshi-related diseases (i.e., CCVDs hypertension, hyperlipidemia, and diabetes mellitus), EFSI could be effective in assessing and preventing the potential risks of Karoshi.

Meanwhile, we should note some limitations of this study. First, we obtained our data in a cross-sectional design, thereby making it difficult to address the causal links. The latest longitudinal study, with 18,700 employees, showed a link between working hours and objectively measured outcomes (i.e., occupational injuries) (Härmä et al. 2020). Therefore, more longitudinal, objective data (especially medical history and working hours) are needed to test the predictability of Karoshi cases being evaluated by the EFSI. Second, our findings have limited generalizability because the targeted occupation in this study was only truck drivers working in Japan. Hence, further research on other risky occupations is required. Third, the 26 excessive fatigue symptoms we examined were derived from 190 investigation reports the listed the prodromes of Karoshi among 1564 compensated reports. Therefore, other important symptoms linking to Karoshi must be collected. Fourth, because the EFSI includes 26 items regarding excessive fatigue symptoms, those items should be scrutinized using a factor analysis on the inventory spread. In relation, more investigations are needed to examine how to rate each symptom. Namely, the grading of the EFSI ranges from 1 (never) to 4 (always) in this study. However, a yes/no question style may be preferable because there could be significant symptoms even if the respondent did not rate a severe grade (e.g., for sudden blindness and sudden unconsciousness).

## Concluding remarks

Our findings suggest that the newly developed Excessive Fatigue Symptom Inventory is effective in evaluating the potential risks of Karoshi. Notably, our data highlight that scheduling working hours to ensure sleep opportunities could be a preventative measure against Karoshi cases among Japanese truck drivers.

## Supplementary Information

Below is the link to the electronic supplementary material.

Supplementary file1 (PDF 110 KB)

## Data Availability

The data obtained in this study are not available because we did not inform the participants of the data transparency nor declare the possibility on the institutional review board.
