# Supplementary material for: Shorter sleep duration is associated with potential risks for overwork-related death among Japanese truck drivers: use of the Karoshi prodromes from worker’s compensation cases
Source: Int Arch Occup Environ Health. 2021 Feb 1;94(5):991–1001. doi: 10.1007/s00420-021-01655-5 (PMC7849961; doi:10.1007/s00420-021-01655-5)
Supplement: Supplementary file 1 — Supplementary file1 (PDF 110 KB) [file 420_2021_1655_MOESM1_ESM.pdf]

Excessive Fatigue Symptom Inventory

In the past 6 months, how often did you experience the following symptoms? Please circle the response that comes closest to how you feel.

		Never	Rarely	Sometimes	Frequently
1	Abnormal sweat	1	2	3	4
2	Severe back and shoulder pain	1	2	3	4
3	Face flushing	1	2	3	4
4	Chest pain and oppressive feeling	1	2	3	4
5	Breathing difficulties	1	2	3	4
6	Repeatedly vomiting	1	2	3	4
7	Heart palpitation	1	2	3	4
8	Numbness of arm and leg	1	2	3	4
9	Sudden blindness	1	2	3	4
10	Heavy headache and dizziness	1	2	3	4
11	Slurring words	1	2	3	4
12	Heavy toothache	1	2	3	4
13	Emotionally arguing with someone (e.g, boss, coworkers, clients, and families)	1	2	3	4
14	Sudden unconsciousness	1	2	3	4
15	Unstoppable nosebleed	1	2	3	4
16	Difficulty in falling asleep at night	1	2	3	4
17	Significant weight loss	1	2	3	4
18	Unrecoverable abnormal fatigue regardless of sleeping or resting	1	2	3	4
19	Abnormal sleepiness	1	2	3	4
20	Becoming short temper	1	2	3	4
21	Losing one's appetite	1	2	3	4
22	Frequently thinking about quitting one's job	1	2	3	4
23	Spending one's days off sleeping	1	2	3	4
24	Going to bed immediately after work due to exhaustion	1	2	3	4
25	Difficulty in awakening from sleep	1	2	3	4
26	Becoming unable to perform daily activities	1	2	3	4
